# Nanoarchitectonics of a Microsphere-Based Scaffold for Modeling Neurodevelopment and Neurological Disease

**DOI:** 10.1021/acsabm.1c01012

**Published:** 2022-01-19

**Authors:** Eric S. Sandhurst, Sharad V. Jaswandkar, Krishna Kundu, Dinesh R. Katti, Kalpana S. Katti, Hongli Sun, Daniel Engebretson, Kevin R. Francis

**Affiliations:** † Department of Biomedical Engineering, University of South Dakota, Sioux Falls, South Dakota 57107, United States; ‡ BioSystems Networks and Translational Research Center, Brookings, South Dakota 57006, United States; § Civil, Construction and Environmental Engineering Department, North Dakota State University, Fargo, North Dakota 58108, United States; ∥ Cellular Therapies and Stem Cell Biology Group, Sanford Research, Sioux Falls, South Dakota 57104, United States; ⊥ Department of Pediatrics, University of South Dakota Sanford School of Medicine, Sioux Falls, South Dakota 57105, United States

**Keywords:** microsphere, scaffold, nanoarchitectonic, three-dimensional, induced pluripotent, iPSC, NSC

## Abstract

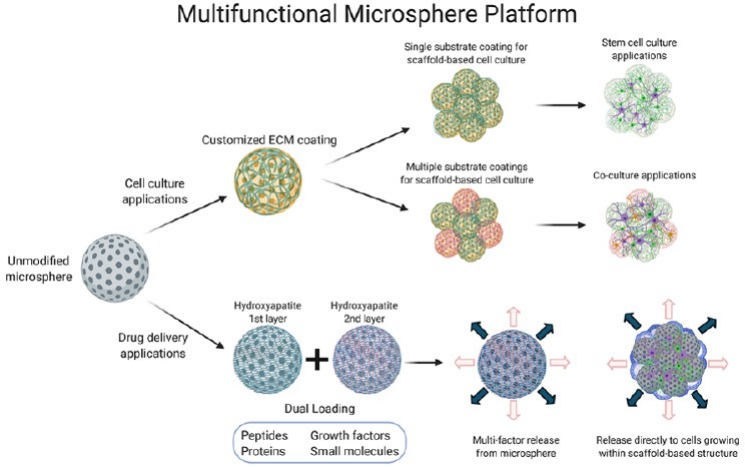

Three-dimensional cellular constructs derived from pluripotent stem cells allow the *ex vivo* study of neurodevelopment and neurological disease within a spatially organized model. However, the robustness and utility of three-dimensional models is impacted by tissue self-organization, size limitations, nutrient supply, and heterogeneity. In this work, we have utilized the principles of nanoarchitectonics to create a multifunctional polymer/bioceramic composite microsphere system for stem cell culture and differentiation in a chemically defined microenvironment. Microspheres could be customized to produce three-dimensional structures of defined size (ranging from >100 to <350 μm) with lower mechanical properties compared with a thin film. Furthermore, the microspheres softened in solution, approaching more tissue-like mechanical properties over time. With neural stem cells (NSCs) derived from human induced pluripotent stem cells, microsphere-cultured NSCs were able to utilize multiple substrates to promote cell adhesion and proliferation. Prolonged culture of NSC-bound microspheres under differentiating conditions allowed the formation of both neural and glial cell types from control and patient-derived stem cell models. Human NSCs and differentiated neurons could also be cocultured with astrocytes and human umbilical vein endothelial cells, demonstrating application for tissue-engineered modeling of development and human disease. We further demonstrated that microspheres allow the loading and sustained release of multiple recombinant proteins to support cellular maintenance and differentiation. While previous work has principally utilized self-organizing models or protein-rich hydrogels for neural culture, the three-dimensional matrix developed here through nanoarchitectonics represents a chemically defined and robust alternative for the *in vitro* study of neurodevelopment and nervous system disorders.

## Introduction

Disorders affecting the nervous system are among the leading causes of comorbidity and death worldwide.^1,2^ Observing and analyzing disease impacts on the nervous system are inherently challenging within affected individuals. The use of model systems to recapitulate different structures and functions of the nervous tissue under study provides a mechanism to study neurological disease. Many of the insights into neuropathological disease have come from research on post-mortem tissue, traditional two-dimensional (2D) cell culture experiments, and animal models such as transgenic mice and rats. Despite the availability of genetic and technological tools and a robust foundation of neuroscience research, these model systems have limitations.^3^ Studying the pathogenesis of complex diseases has proven to be particularly difficult because of a lack of access to healthy and diseased brain tissue, immature and spatially limited *in vitro* cell culture systems, and animal models that fail to capture the developmental, architectural, and species-specific aspects of the human brain.^2,4^ Therefore, additional models of the human nervous system are needed to help overcome some of these limitations.

Human induced pluripotent stem cells (iPSCs) have created a fundamental shift in how scientists study human disease. By establishing a reliable method for generating individual-specific pluripotent cells, iPSCs represent a robust model system for the study of human disease and may accelerate progress toward revolutionary treatments.^5^ iPSC-derived neural stem cells (NSCs) are therefore a useful tool to provide insights into the underlying mechanisms of neurodevelopment and neurodegenerative diseases. The use of iPSCs has led to new strategies for therapeutic intervention and increased accuracy for drug discovery. Although iPSCs represent a revolution in studying development and human disease *in vitro*, researchers have predominantly relied on 2D culture platforms.^6^ Since traditional monolayer cultures support only planar cell–cell interactions, these systems poorly simulate the natural three-dimensional (3D) microenvironment of the body. The natural interaction and communication between the heterogeneous milieu of cells and the extracellular matrix found within the body are difficult to replicate in 2D culture.^7^ Certain cellular characteristics, including apicobasal polarity and guided cell migration, cannot be recapitulated in planar culture systems.^8^ Spatially complex iPSC models of neurological disease are thus needed.^9^

As recent groundbreaking studies have shown, 3D culture of iPSCs more accurately represents the spatial arrangement and temporal development of nervous tissue compared with 2D models.^3,10^ Research conducted with 3D culture models provides new knowledge of areas that were previously only poorly modeled or inaccessible altogether, such as the cerebral cortex, neocortex, ventral forebrain, ventral telencephalon, cerebellum, midbrain, choroid plexus, and optic cup.^10−13^ Although each 3D protocol has advantages and disadvantages, they all utilize the capacity of embryonic stem cells or iPSCs to self-organize, self-assemble, and differentiate within a 3D environment.^2^ Known as spheroids, neurospheres, cellular scaffolds, or organoids depending on their complexity and the methods used, these 3D platforms can produce functional, highly organized populations of cells.^1,14^ However, 3D models are still limited by experimental heterogeneity, limited control over tissue organization, inadequate diffusion and heterogeneous distribution of macromolecules, and end point analyses.^15−17^

To help overcome the limitations of current 3D models, we have developed a microsphere-based scaffold with nanoarchitectural features for iPSC-based neural differentiation.^18,19^ Using a biomaterial-based microenvironment, we have created an alternative to the undefined components present within other materials-based 3D culture systems. We have defined the mechanical properties of this scaffold, demonstrated the maintenance and lineage differentiation of iPSC-derived NSCs cultured on the scaffold, established a protocol for coculture of multiple neural and endothelial cell types, and utilized this scaffold for localized cellular delivery of small molecules. This system represents a novel advancement in 3D culture and provides a multifunctional platform for disease modeling, drug screening applications, and developmental studies.

## Experimental Section

### Chemicals and Reagents

Poly(lactic-*co*-glycolic acid) (PLGA) (50:50, 1.15 dL/g) was purchased from Lactel (Birmingham, AL). Gelatin type A, dichloromethane (DCM), poly-l-ornithine (PLO), molecular-grade water, bovine serum albumin (BSA), disodium ethylenediaminetetraacetate (EDTA), and magnesium chloride were purchased from Sigma-Aldrich (St. Louis, MO). Low-attachment 24-well plates, sodium chloride, sodium bicarbonate, Tris base, Neurobasal medium, and epidermal growth factor were purchased from Thermo Fisher Scientific (Carlsbad, CA). B27 supplement with vitamin A, B27 without vitamin A, Accutase and GlutaMAX were all purchased from Life Technologies (Carlsbad, CA). Basic fibroblast growth factor (bFGF) was purchased from Reprocell (Beltsville, MD). Y27632 ROCK inhibitor was purchased from Reagents Direct (Encinitas, CA). mTeSR1 was purchased from Stem Cell Technologies (Vancouver, BC). DMEM, DMEM-F12, penicillin/streptomycin, One Shot fetal bovine serum (FBS), trypsin-EDTA, and phosphate-buffered saline (PBS) were purchased from Gibco (Carlsbad, CA). Brain-derived neurotrophic factor (BDNF) and glial cell line-derived neurotrophic factor (GDNF) were purchased from Peprotech (Rocky Hill, NJ). Matrigel hESC-Qualified Matrix was purchased from Corning (Glendale, AZ). Laminin was purchased from Invitrogen (Carlsbad, CA). Hydrochloric acid was purchased from Avantor Performance Materials (Center Valley, PA). Poly(vinyl alcohol) (PVA) was purchased from PolySciences, Inc. (Warrington, PA). Ethanol, calcium chloride, and sodium phosphate were purchased from Acros Organics (Fair Lawn, NJ). Ultralow-attachment 96-well plates were purchased from Nexcelom Bioscience (Lawrence, MA).

### Preparation of Microspheres

A double emulsion procedure was used to prepare porous microspheres. First, 0.5 g of 50:50, 1.15 viscosity PLGA was placed into a glass vial with 15 mL of DCM. PLGA was dissolved under constant stirring at 700 rpm at 50 °C. Simultaneously, the primary aqueous phase was prepared by dissolving 0.4 g of type A porcine gelatin and 5 mg of PVA in 5 mL of deionized (DI) water in a separate glass vial. A third solution, the secondary aqueous phase, was prepared by dissolving 200 mg of PVA in 200 mL of DI water and cooled to 4 °C. The dissolved polymer solution was poured into a 25 mL beaker and placed on a hot plate at 50 °C under an IKA homogenizer (IKA Works, Inc., Wilmington, NC). The aqueous solution was added manually using a 1000 μL pipet, and the two solutions were emulsified for 5 min at 4000 rpm. The primary emulsion was immediately poured into the secondary aqueous phase and rotated using a magnetic stir plate at 400 rpm for 60 min. After 60 min of stirring at 400 rpm, the contents of the beaker were poured into 1200 mL of fresh DI water and stirred overnight at 300 rpm to facilitate DCM evaporation. The supernatant was discarded, and the microspheres were rinsed, collected in a 50 mL conical tube, kept at −80 °C for 60 min, and lyophilized for 36–48 h. Following lyophilization, the microspheres were treated with an ethanolic sodium hydroxide solution at a ratio of 20% 1 M NaOH and 80% pure ethanol^20^ and then placed into a 50 mL conical tube and vortexed for 20–30 s. The microspheres were rinsed with DI water, collected in a nylon cell strainer, kept at −80 °C for 60 min, and lyophilized for 36–48 h.

### Deposition of Hydroxyapatite on Microspheres

The process for mineralization of PLGA microsphere scaffolds was performed as previously published.^21^ Briefly, the microspheres were divided into fractions on the basis of diameter (*e.g.*, 150–300 μm) by filtering them through ATSE metal sieves of decreasing size. Hydroxyapatite (HA) was formed on the entire exposed surface of the microsphere structure during two phases of immersion into two solutions known as simulated body fluid (SBF). First, microspheres were immersed into a phase I nucleation solution (P1). For P1, 19.95 g of NaCl followed by 0.69 g of CaCl_2_, 0.45 g of Na_2_HPO_4_, 0.88 g of NaHCO_3_, and 0.76 g of MgCl_2_ were dissolved in 500 mL of DI water under stirring conditions. After 150–300 μm diameter microspheres (25 mg) were placed into a glass vial, 25 mL of P1 nucleation solution was added to the vial. Each vial was placed into an orbital shaker, heated to 37 °C, and set for 100 rpm for 12 h. To verify P1 deposition, a FITC-labeled scrambled peptide (FITC-QEQLERALNSS, Biomatik) was added to the P1 SBF and imaged by confocal microscopy.^22^

After 12 h, the microspheres were collected in a nylon cell strainer, kept at −80 °C for 60 min, and lyophilized for 18–24 h. Next, a phase II propagation solution (P2) was created by dissolving various salts. For P2, 0.27 g of CaCl_2_ followed by 3.98 g of NaCl and 0.175 g of Na_2_HPO_4_ were dissolved in 497.5 mL of DI water and 2.5 mL of 10 M HCl under stirring conditions. Tris buffer was added to achieve a pH of 7.4. P1 microspheres were placed in a new glass vial, and 25 mL of P2 propagation solution was added to the vial. Each vial was placed into an orbital shaker, heated to 35 °C, and set for 100 rpm for 12 h. The microspheres were then collected in a nylon cell strainer, kept at −80 °C for 20 min, and lyophilized for 18–24 h. To verify P2 deposition, BSA conjugated to Alexa Fluor 647 (Invitrogen, Carlsbad, CA) was added to the P2 SBF and imaged by confocal microscopy.

### Poly-l-ornithine and Laminin Coating of 2D and 3D Surfaces

PLO (0.2% v/v) diluted in molecular-grade water was added to culture surfaces and allowed to conjugate for 12 h in a 37 °C incubator. The dishes were rinsed twice with molecular-grade water before a 1% v/v solution of natural mouse laminin diluted in PBS was added to each well. Culture dishes were incubated at 37 °C for 12 h and either used immediately or stored at −20 °C. The microspheres were immersed in 0.2% v/v PLO and placed in an enclosed orbital shaker maintained at 37 °C and 100 rpm for 12 h. Then the microspheres were rinsed twice with molecular-grade water, placed into a new glass vial, immersed in a 1% solution of natural mouse laminin, and placed in an enclosed orbital shaker set for 37 °C and 100 rpm for 12 h. PLO+laminin-coated microspheres were kept at 4 °C and used within 12 h.

### Ultrastructural Characterization of Microspheres

An FEI Quanta 450 field-emission scanning electron microscope (SEM) was used to characterize the morphological structures of microsphere samples. Overall microsphere diameter was analyzed using SEM images. Micro computed tomography (micro CT) was performed by ScanCo Associates, (ScanCo μCT 50, Brüttisellen, Switzerland) to measure the local pore diameter. The microsphere porosity was calculated from micro CT imaging, performed by ScanCo Associates, using the following equation:
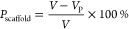
where *P*_scaffold_ is the porosity of the microsphere batch, *V* is the total volume of the microsphere batch, and *V*_P_ is the volume of PLGA, given by the mass divided by the density of PLGA (ρ = 1.3 g/cm^3^).

### Nanomechanical Evaluation of Microspheres

To prepare a PLGA film for mechanical testing, 0.5 g of 50:50 PLGA (3.3% w/v), 0.75 g of 50:50 PLGA (5% w/v), and 1 g of 50:50 PLGA (6.6% w/v) were each dissolved in 15 mL of DCM and poured into a 25 mL glass beaker. Once the solvent evaporated, testing coupons were cut from each film and attached to titanium metal sections (10 mm × 10 mm × 0.25 mm) (Sigma, St. Louis, MO) with 100 μL of Elmer’s glue (Westerville, OH). To prepare PLGA microsphere samples for nanomechanical testing, 100 μL of Minwax polyacrylic (Upper Saddle River, NJ) was first applied to titanium sections using a spin-coating system. A Dremel rotary tool (Dremel, Racine, WI) was used at 10 000 rpm for 5 s to obtain a uniform polyacrylic layer before adherence of microspheres or films to the substrates was achieved. The samples were allowed to dry completely. Prior to nanoindentation experiments, some samples were rehydrated in Neurobasal medium for 1, 2, or 7 days. Samples were removed from the aqueous phase and carefully blotted before nanoindentation.

A Hysitron Triboindenter nanoindenter (Hysitron Inc., Minneapolis, MN) with a pyramidal Berkovich diamond indenter tip (tip radius of 200 nm) was used to calculate the mechanical properties of three PLGA films (3.3, 5, and 6% w/v) and PLGA microspheres (3.3% w/v) in the dry and hydrated states. After calibration with a standard fused quartz reference sample, an indentation depth was set at 1000 nm with a 20 nm/s displacement rate. The elastic modulus (*E*) and indentation hardness (*H*_IT_) of each sample were measured at room temperature. A displacement depth of 1000 nm was selected for all quasistatic nanoindentation experiments, resulting in reliable elastic property measurements free of substrate effects. Average values of *E* and *H*_IT_ were calculated from the analysis of 30 unique microspheres. The estimation methods for determining *E* and *H*_IT_ were based on the methods of Oliver and Pharr.^23−26^ These methods have been applied to make direct nanoindenter-based measurements of elastic and inelastic properties of soft materials such as human cells.^27,28^

### Fourier Transform Infrared Spectroscopy (FTIR) Analysis

Transmission FTIR spectroscopy studies were performed using samples of PVA, gelatin, PLGA, microspheres, microspheres coated with HA for 12 h, and microspheres coated with HA for 24 h. Samples were sandwiched between two KBr windows and placed in a universal sample holder. A Thermo Nicolet Nexus 870 spectrometer equipped with a KBr beamsplitter was used to perform these experiments in the range of 4000–960 cm^–1^. A spectral resolution of 4 cm^–1^ and 32 scans were used for each sample.

### Culture of Human iPSCs and NSCs and Neural Differentiation

Two control human iPSC lines, NL5 (NCRM-5) (a kind gift from the iPSC Core Facility, NHLBI, Bethesda, MD) and Scui21 (Scui) (a kind gift from the NIH Stem Cell Unit, NINDS, Bethesda, MD), and one Smith–Lemli–Opitz syndrome (SLOS) patient-derived iPSC line (CWI 4F2; a kind gift from Dr. Forbes Porter, NICHD, Bethesda, MD) were cultured and directed toward NSCs using a rosette-based assay as previously published.^29,30^ Following their derivation and expansion, NSCs were cultured on PLO+laminin-coated 35 mm tissue culture dishes in NSC medium (DMEM, 2 mM glutamine, B27 minus vitamin A, 20 ng/mL EGF, 20 ng/mL bFGF, 50 μg/mL penicillin–streptomycin) supplemented with ROCK inhibitor Y27632 (10 μM). The medium was changed every other day. The cells were passaged *via* incubation with Accutase at 37 °C for 3–5 min. The enzymatic reaction was stopped by addition of Y27632 to the NSC culture medium followed by centrifugation at 1500 rpm. The cells were divided evenly between two new PLO+laminin-coated culture dishes (approximately 2.5–3 × 10^6^ cells per dish).

To induce neural differentiation, NSCs were collected from 35 mm cell expansion dishes as described above. Upon resuspension in NSC medium, the cells were plated in 24- or 96-well plates coated with PLO+laminin (Thermo Fisher Scientific, Waltham, MA) or Lab-Tek Nunc four-well chamber glass slides coated with PLO+laminin. Cells were maintained in NSC medium supplemented with 10% FBS for 4 days and then changed to neural differentiation medium (Neurobasal medium, B27 with vitamin A, 10 ng/mL GDNF, 10 ng/mL BDNF, 2 mM glutamine, 50 μg/mL penicillin–streptomycin) for the duration of differentiation. For neurosphere culture, NSCs were collected *via* Accutase and plated at 150 000 cells/well in an ultralow-attachment round-bottom 96-well plate. Neurospheres were maintained in suspension in NSC medium supplemented with FBS for 4 days and then changed to NSC differentiation medium for the duration of each experiment. For microsphere culture of NSCs, microspheres (100 μg) were added to each round-bottom well of an ultralow-attachment 96-well plate. Upon resuspension in NSC medium, 150 000 cells were passively seeded onto the microspheres. The microspheres were cultured in NSC medium supplemented with FBS for 4 days and then changed to neural differentiation medium for the duration of each experiment. All of the culture plates and dishes were cultured at 37 °C with 5% CO_2_.

### Impact of Serum on NSC Microsphere Attachment

Microspheres were immersed in 70% ethanol for 60 min on an orbital shaker set at 100 rpm. Microspheres (1 mg) were transferred to a low-attachment flat-bottom 24-well plate before NSCs were seeded onto the microspheres (150 000 cells per 1 mg of microspheres per well) in NSC medium. Serum-supplemented groups received 10% FBS. The cells/scaffolds were cultured in a 24-well plate at 37 °C with 5% CO_2_.

### Evaluation of Substrates for NSC Microsphere Attachment

Microspheres were divided into fractions and sterilized as previously mentioned. The microspheres were coated with PLO+laminin as above. Microspheres receiving a Matrigel coating were placed into a sterile glass vial and incubated in either Matrigel for 2 h on an orbital shaker set for 50 rpm at room temperature. Uncoated or substrate-coated microspheres were transferred to the wells of a 24-well plate before NSC control line cells were seeded into the scaffold (150 000 cells per 1 mg of microspheres). Cells/scaffolds were cultured in a 24-well plate at 37 °C with 5% CO_2_.

### Astrocyte Generation from iPSC-Derived NSCs

NSCs (70 000) were plated onto 35 mm PLO+laminin-coated tissue culture dishes and maintained in neural differentiation medium at 37 °C with 5% CO_2_ through day 14. On day 14, cells were collected *via* Accutase and transferred to 35 mm tissue culture plates coated with 25 μg/mL poly-d-lysine (PDL) (Sigma-Aldrich, St. Louis, MO), and the medium was changed to astrocyte differentiation medium (DMEM/F12, 2 mM glutamine, 10% FBS, and 1% penicillin–streptomycin). The medium was changed every 48 h through day 28. On day 28, the cells were collected with 0.25% Trypsin-EDTA and transferred to a PDL-coated T25 tissue culture flask for expansion. Astrocytes were expanded and passaged with Trypsin-EDTA for an additional 30–45 days as needed prior to use.

### Human Umbilical Vein Endothelial Cell (HUVEC) Microsphere Culture

HUVECs obtained from Lonza (Walkersville, MD) were plated on T25 flasks and cultured with Endothelial Basal Medium-2 (cat no. 00190860, Lonza, Walkersville, MD) at 37 °C with 5% CO_2_.^22^ HUVECs were harvested from the flask by rinsing with PBS, addition of 2 mL of 0.05% Trypsin-EDTA to the flask, and incubation at 37 °C for 3–5 min. The cells were centrifuged at 1500 rpm for 5 min, after which the supernatant was aspirated and the cell pellet was resuspended in neural differentiation medium before addition to microspheres.

### Multilineage Coculture Using Microsphere Scaffolds

Microsphere samples were immersed in 70% ethanol for 60 min on an orbital shaker set at 100 rpm. Microspheres (100 μg) were added to each well of an ultralow-attachment 96-well plate. On day 0, NSCs were collected from 35 mm cell expansion dishes *via* Accutase as described above. Upon resuspension, NSCs were passively seeded onto 100 μg of microspheres. On day 2, astrocytes were passively seeded onto the NSC-only microspheres. On day 5, HUVECs were added to each NSC+astrocyte scaffold. All of the groups were cultured in NSC differentiation medium at 37 °C with 5% CO_2_.

### Immunofluorescent Imaging of Scaffold-Cultured Cells

The cell lineage of differentiating NSCs was visualized by immunofluorescence using primary and secondary antibodies. Cell-based spheroids and cell-seeded microspheres were fixed in 4% paraformaldehyde for 20 min, rinsed with 1× PBS, and permeabilized with 0.1% TritonX-100 for 20 min. Samples were subsequently blocked with 5% BSA containing 0.1% TritonX-100 in PBS for 60 min before the following primary antibodies were added: chicken anti-GFAP (Novus Biologicals, NBP1-05198, 1:2000), mouse anti-βIII-tubulin (Millipore, MAB1637, 1:1000), mouse anti-human Nestin (Millipore, MAB5326, 1:2000), mouse anti-MAP2 (Synaptic Systems, 188 011, 1:2000), rabbit anti-Neurofilament, medium-chain (Novus Biologicals, NB300-133, 1:2000), rabbit anti-SOX2 (Cell Signaling, 3579S, 1:400), mouse anti-Ki67 (Abcam, ab15580, 1:2000), or mouse anti-CD31 (Abcam, ab9498, 1:1000). The samples were incubated with primary antibody at 4 °C overnight. Following overnight incubation, the samples were incubated for 60 min with the following secondary antibodies diluted in blocking buffer: Alexa Fluor 555 rabbit anti-mouse IgG (Life Technologies, A21427), Alexa Fluor 555 donkey anti-rabbit IgG (Life Technologies, A31572), Alexa Fluor 488 goat anti-mouse IgG (Life Technologies, A11001), Alexa Fluor 488 goat anti-rabbit IgG (Life Technologies, A11008), or Alexa Fluor 488 goat anti-chicken IgG (Life Technologies, A11039). All secondaries were diluted 1:500. After rinsing, Fluoromount-G with 4′,6-diamidino-2-phenylindole (DAPI) was added. The samples were imaged using a confocal laser scanning microscope (Olympus Fluoview FV1200, Olympus, Japan).

### Hematoxylin and Eosin Staining of Scaffold Cultures

The scaffolds were fixed in 10% neutral buffered formalin and processed on a Leica 300 ASP tissue processor. The tissues were embedded in paraffin and serially sectioned at 5 μm thickness. Slides were stained with hematoxylin and eosin (H&E) on a Sakura Tissue-Tek automated H&E staining instrument. The program runs as follows: deparaffinize and rehydrate tissue, stain in Gill’s III hematoxylin, differentiate with running tap water, blue in ammonia–water, counterstain in eosin, and dehydrate and clear. All of the images were taken on a Nikon NiE microscope using a Nikon DS-Fi2 camera and a 20×/0.75 PlanApo λ objective.

### BSA Loading and Release from Microspheres

Microspheres were coated with HA as discussed above with minor changes. BSA (2.5 mg) was added to 25 mL of SBF in each combination (+P1–P2; −P1+P2; +P1+P2) and incorporated into the HA. When the microspheres were collected after each HA deposition phase, the supernatant was saved to analyze the BSA remaining in the solution. The microspheres were also rinsed with 1 mL of DI water, and the rinse solution was saved to calculate the incorporation efficiency. To measure the amount of BSA incorporated into the HA microspheres, four groups of BSA-loaded microspheres were immersed in 0.5 M EDTA solution, vortexed for 1 min, and centrifuged at 2000*g* for 2 min. The incorporation efficiency was determined by calculating the BSA remaining in the SBF supernatant, the BSA in the rinse solution, and the BSA released from the microspheres. To model release, BSA–HA microspheres (10 mg) were added to microcentrifuge vials with 1 mL of PBS and placed into an incubating shaker set for 100 rpm and 37 °C. At predetermined time points (30 min, 1 h, 2 h, 5 h, 12 h, day 1, day 2, day 3, day 7, day 10, and day 15), 500 μL of PBS eluent was removed and 500 μL of fresh PBS was added to the tube. Analysis of BSA release was performed using a Pierce bicinchoninic acid (BCA) protein assay kit (Thermo Fisher Scientific, Waltham, MA) per manufacturer’s instructions.

### bFGF Loading, Release, and Impact on Cell Viability

Microspheres were coated with HA as previously discussed. bFGF (20 ng/mL) was added to both SBF phases (+P1+P2) and incorporated into the crystal matrix. NSCs were passively seeded onto 100 μg of microspheres. The microsphere-based scaffolds were cultured in NSC medium for 14 days at 37 °C with 5% CO_2_. At each time point (day 1, day 4, day 7, and day 14), bFGF–HA scaffolds were analyzed by MTS assay to determine the amount of proliferation compared with other 2D and 3D groups. Each group was cultured in triplicate, and 50% of the cell culture medium was replenished every 48 h. Cell viability was quantitatively analyzed using the CellTiter 96 Aqueous One Solution Cell Proliferation Assay (MTS, Promega, USA) according to the manufacturer’s instructions. In brief, after culturing for 1, 4, 7, or 14 days in ultralow-attachment round-bottom 96-well plates, the culture medium was removed, fresh medium with 10% MTS solution was then added, after which the plates were incubated at 37 °C with 5% CO_2_ in the dark for 1 h. Each biological replicate was analyzed in quadruplicate by removal of 100 μL volumes from each well. The absorbance was measured at 490 nm using a microplate reader (Infinite M200, Tecan, USA). Cell viability was expressed as the number of cells calculated from the slope of a standard curve prepared by culturing NSCs at densities from 50 000 to 500 000 on PLO+laminin-coated wells of a 24-well plate (data not shown).

### Statistical Analyses

To determine the statistical significance of the observed differences between the study groups, a two-tailed Student’s *t* test was applied to the control group and each experimental group. A value of *p* < 0.05 was considered to be statistically significant. Values are reported as the mean ± one standard deviation (SD). Microscopy images across treatments were imaged using equivalent laser power and exposure times.

### Human Subjects Research Statement

All research performed using human cell lines was determined not to constitute human subjects research by the institutional review board of Sanford Research.

## Results

### Preparation of a Microsphere Scaffold for Culture of iPSC Derivatives Is Rapid and Tunable

**Figure 1:**
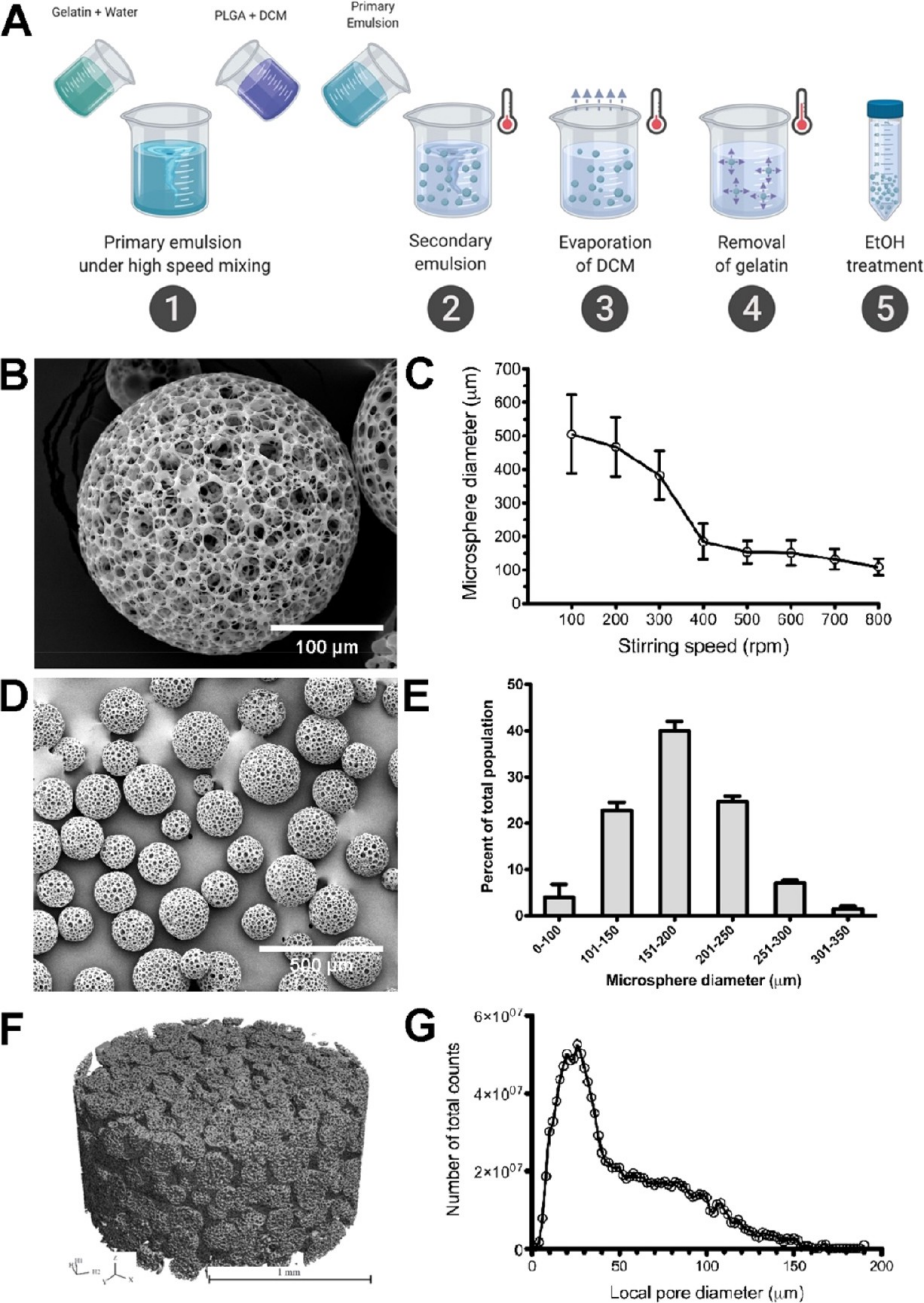
Preparation and characterization of the microsphere scaffold. (A) Illustration of the double emulsion and porogen leaching process used to prepare porous PLGA microspheres. (B) SEM image of a single porous microsphere (scale bar = 100 μm). (C) The defined stirring speed during secondary emulsion dramatically impacted the mean microsphere diameter (*n* = 3 per treatment, each group contained 250 microspheres). (D) SEM image of a representative batch of microspheres (scale bar = 500 μm). (E) Distribution of microsphere size across multiple batch preparations using a stirring speed of 400 rpm (*n* = 3 biological replicates, each replicate contained 250 microspheres). (F) Micro CT image of the internal microsphere structure (scale bar = 1 mm). (G) Local pore diameter as calculated by micro CT. Error bars represent ±1 standard deviation.

To create a scaffold for culture of iPSC derivatives, we utilized a double emulsion and porogen leaching technique to yield a highly uniform poly(lactic-*co*-glycolic acid) (PLGA) microsphere matrix with interconnected pores and >88% overall porosity. Gelatin was utilized as the sacrificial porogen to create spherical pores within the PLGA matrix. Through optimization of each step within the preparation process, we have created a stable, consistent microsphere structure (Figure 1A). FTIR analysis of the various materials utilized for microsphere generation and coating was performed to verify production material chemistries in comparison to spectra within the final microsphere product (Figure S1). The spectra indicate nonbonded interactions between the hydroxyapatite and polymers. Through variations in the speed of mixing the gelatin/PLGA during the emulsion process, we were able to control the microsphere diameter (Figure 1B,C). With a 400 rpm mixing step, the microsphere diameter exhibited reduced variability, and the majority remained within the 100–250 μm range (Figure 1D,E). On the basis of the mean microsphere diameter achieved, we utilized a mixing speed of 400 rpm for all subsequent microsphere assays. Microspheres were packed into a micro CT chamber with a volume of 3.14 mm^3^ (Figure 1F). Analysis revealed a local pore diameter of 50 ± 35 μm, >88% porosity, and an open, interconnected pore structure (Figure 1G). The process described in this study optimizes the microsphere porosity, size distribution, and reproducibility for use as a scalable platform for 3D cell culture applications.

### The Mechanical Properties of Microsphere Scaffolds Are Impacted by Hydration

**Figure 2:**
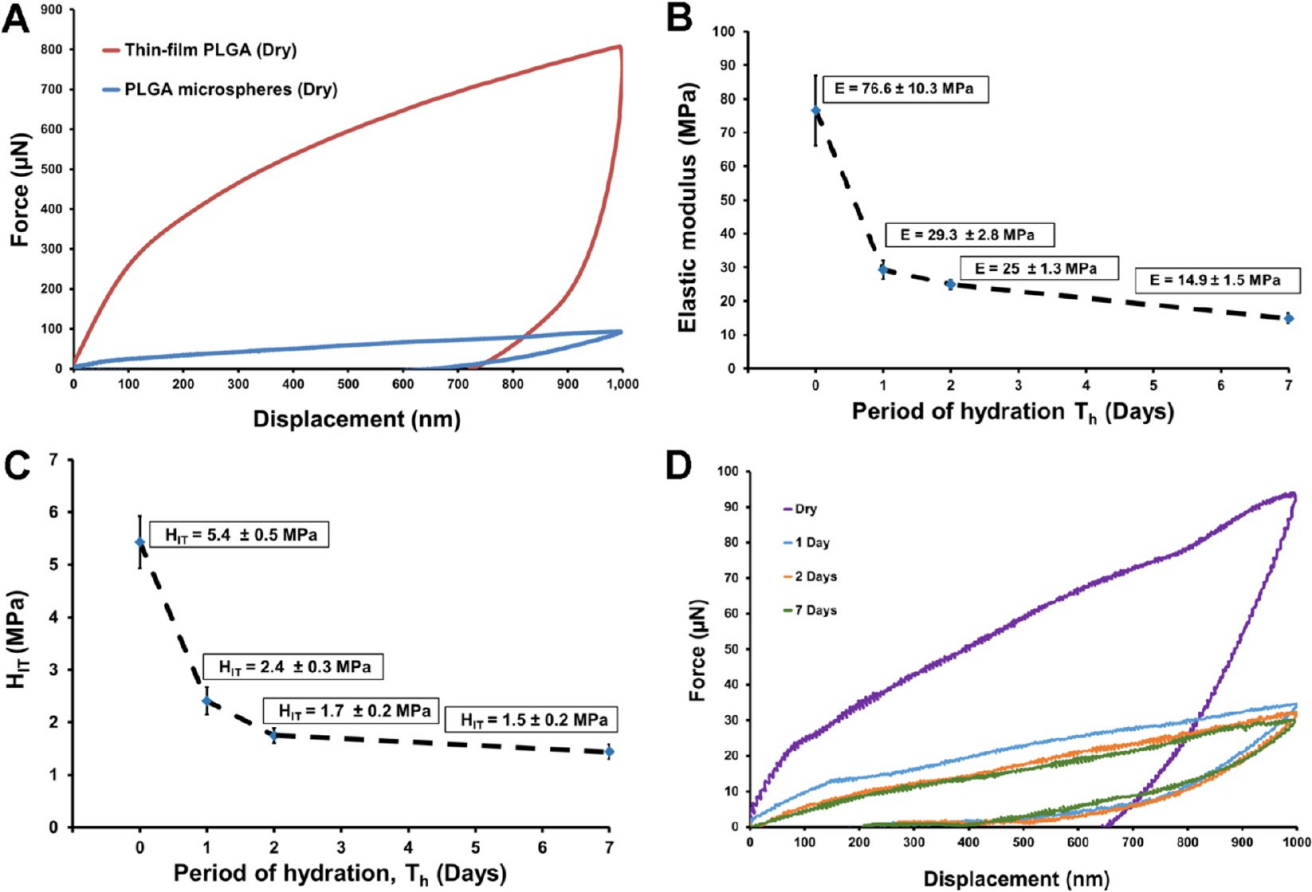
Hydration of the microsphere scaffold (3.3% w/v) shifts the load–displacement curves, elastic modulus, and indentation hardness as functions of time. (A) Load–displacement response for the PLGA thin film and microspheres in the dry state demonstrates the softening effect of the porous microstructure of the microspheres. (B–D) Deformation response and mechanical properties of the hydrated PLGA microspheres compared with the dry state with degradation. All error bars for elastic modulus measurements (B) and indentation hardness (C) represent ±1 standard deviation.

Nanoindentation assays were performed to determine the mechanical properties of PLGA samples. The nanomechanical properties of a PLGA thin film (dry state) and microspheres (both dry and hydrated states) were determined as a function of the period of hydration (*T*_h_). The load–displacement responses for the PLGA thin film and microspheres were measured in displacement-controlled loading and unloading mode. We determined the average elastic modulus for nonhydrated PLGA thin films of 3.3, 5, or 6.6% w/v to be *E* = 1.48, 0.619, and 0.129 GPa, respectively. The indentation hardness (*H*_IT_) obtained for these films equated to 34.6 ± 2.4, 15 ± 1.1, and 6.2 ± 0.4 MPa for 3.3%, 5%, and 6.6% w/v nonhydrated PLGA films, respectively. By comparison, the elastic modulus and indentation hardness values for nonhydrated PLGA microspheres (3.3% w/v) were significantly lower (*E* = 76.6 ± 10 MPa and *H*_IT_ = 5.4 ± 0.5 MPa, respectively) than those for the nonhydrated thin film (3.3% w/v), demonstrating the mechanical impact of the porous architecture (Figure 2A). The force–displacement response from PLGA microsphere indentation captures the microstructural response of both the PLGA polymer structure and the pore spaces. The highly porous microspheres produced significantly lower mechanical properties compared with the film. The elastic modulus and indentation hardness of the PLGA microspheres (3.3% w/v) decreased as the hydration increased (Figure 2B,C). For hydrated microspheres, a nearly 40% decrease in modulus (Figure 2B) was observed after 24 h of hydration relative to the dry state (*E* = 76.6 ± 10 MPa). The modulus dropped to *E* = 29.3 ± 2.8, 24.9 ± 1.3, and 14.9 ± 1.5 MPa on day 1, day 2, and day 7, respectively. The hardness values also decreased similarly with increased hydration (Figure 2C). While *H*_IT_ = 5.4 ± 0.5 MPa in the dry state, *H*_IT_ decreased over time with prolonged hydration (*H*_IT_ = 2.4 ± 0.3, 1.7 ± 0.1,a and 1.44 ± 0.2 MPa on day 1, day 2, and day 7, respectively). A comparison of load-displacement curves for microspheres in dry versus hydrated states demonstrates that for the same maximum dispacement value, peak load emerged as a function of the hydration period. Hydrated PLGA microspheres displayed significant elastic recovery upon unloading (Figure 2D). These data demonstrate that our PLGA-based scaffold exhibits mechanical properties that become more tissue-like with incubation in aqueous solutions such as culture medium.

### Optimization of iPSC-Derived NSC Scaffold Attachment

**Figure 3:**
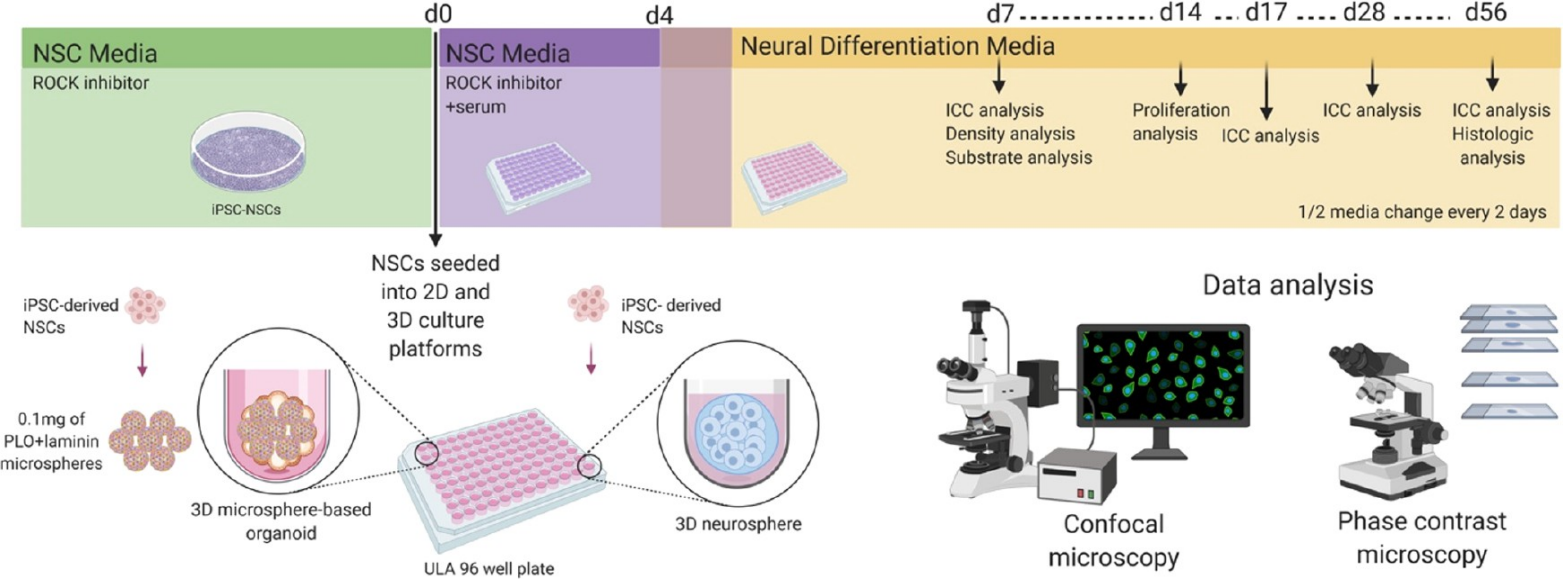
Assay schema for validating the use of a PLGA-based microsphere system for neural cell models. iPSC-derived NSCs were either cultured in traditional 2D systems, grown as self-aggregating 3D neurospheres, or seeded onto 3D-microsphere-based structures. Cultures were then analyzed for various cellular parameters, including attachment, proliferation, differentiation, and coculture.

**Figure 4:**
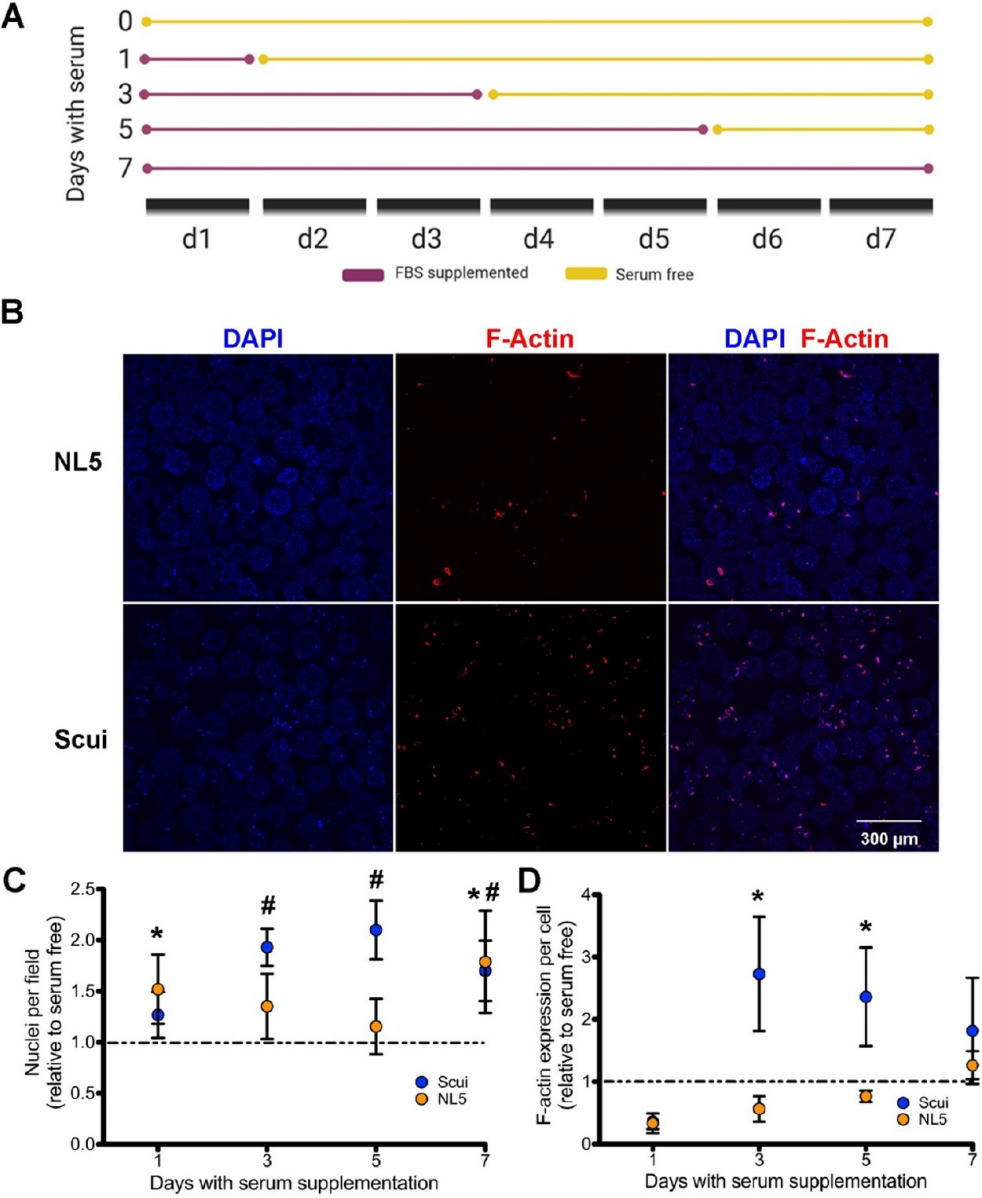
Serum improves attachment and cytoskeleton production by microsphere-cultured NSCs. (A) Diagram depicting the experimental design for serum-supplemented medium exposure. (B) Confocal images of NSCs on uncoated microspheres after 7 days of serum supplementation. Nuclei are identified with DAPI, and F-actin filaments are labeled with phalloidin–Texas Red (scale bar = 300 μm). (C) Nuclei counts of NSCs after various durations of serum supplementation (*n* = 3 biological replicates per group). (D) F-actin per cell quantified by phalloidin–Texas Red after various durations of serum supplementation (*n* = 3 biological replicates per group). Error bars represent ±1 standard deviation. * indicates a significant increase (*p* < 0.05) in Scui NSCs compared with the serum-free control; # indicates a significant increase (*p* < 0.05) in NL5 NSCs compared with the serum-free control.

We next sought to determine whether our newly developed PLGA-based material could function as a cellular scaffold and model for neurodevelopment. Beginning with the addition of iPSC-derived NSCs to the scaffold, we outlined a series of assays to qualify the ability of our PLGA-based material to promote NSC attachment, proliferation, and differentiation and support coculture studies (Figure 3). To first determine the efficiency of iPSC-derived NSC attachment onto our PLGA microsphere surface, NSCs were cultured with unmodified PLGA microspheres in the presence or absence of FBS for 1, 3, 5, or 7 days (Figure 4A). The addition of serum has previously been shown to aid in the attachment of neural cell types to culture matrixes.^31−35^ Through balancing the positive impact on NSC attachment while minimizing the influence of FBS on neural differentiation, neural differentiation and tissue modeling could be optimized. NSCs were passively seeded onto unmodified PLGA microspheres in the presence or absence of FBS and cultured for 7 days before being fixed for immunocytochemistry (ICC) (Figure 4A). Analysis of two distinct NSC lines revealed that serum supplementation for any length of time increased the number of nuclei per microsphere compared with the non-FBS-supplemented case (Figure 4B,C). Additionally, F-actin expression, as a measure of cytoskeleton formation, was increased by NSC serum supplementation (Figure 4D). While these data suggest that short-term exposure to serum increases NSC microsphere attachment, substrates that avoid the inhibitory effects of serum on neural differentiation may benefit NSC properties.^36^

**Figure 5:**
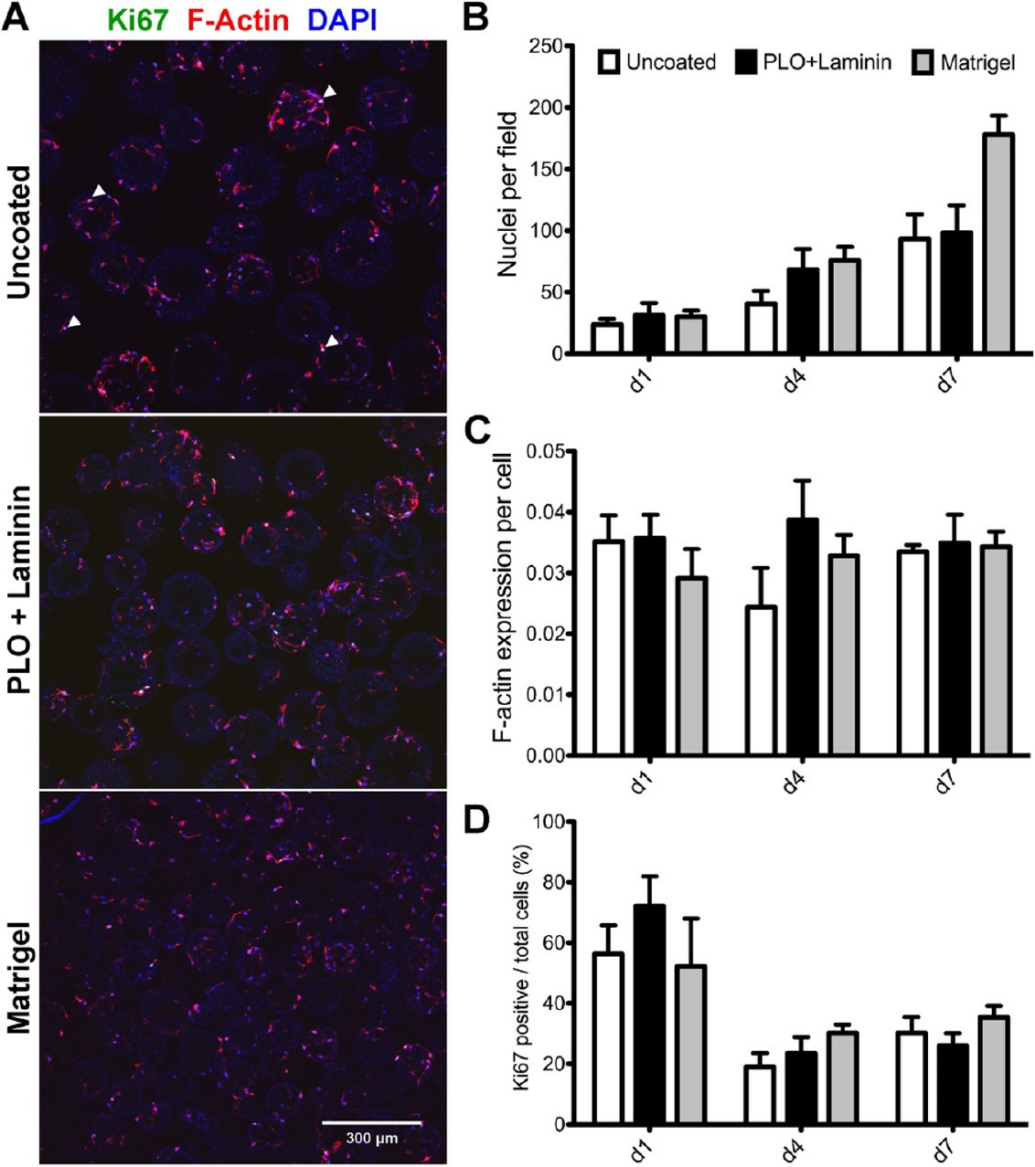
Neural-supportive substrates promote proliferation and cytoskeletal production from microsphere-cultured NSCs. (A) Confocal images of Scui NSCs at day 7 on uncoated microspheres (top panel), PLO+laminin-coated microspheres (middle panel), and Matrigel-coated microspheres (bottom panel). Scale bar = 200 μm. Arrowheads indicate selected Ki67-positive cells. (B) Increasing cell counts on uncoated, PLO+laminin-coated, and Matrigel-coated microspheres over 7 days. (C) The volume of F-actin per cell on day 7 remained constant despite increasing cell number. (D) No significant difference between the percentages of Ki67-positive cells for uncoated and coated microspheres was observed (*n* = 15, three biological replicates and five image fields per group). Error bars represent ±1 standard deviation.

Through serum-free culture of embryoid-body-like aggregates with quick reaggregation (SFEBq), *in vitro* neuronal differentiation can be achieved in the absence of extrinsic neural induction factors.^3,37^ It is also established that growth factor and protein-rich hydrogels such as Matrigel support the development of 3D neural cultures.^38^ Since our biomaterial-based methodology supports 3D self-organization and the minimization of undefined factors, we compared the responses of NSCs cultured on uncoated microspheres to those of microspheres coated with two different neural supportive substrates: PLO+laminin and Matrigel. Confocal microscopy images demonstrated that NSCs attached to either uncoated, PLO+laminin-coated, or Matrigel-coated microspheres (Figure 5A). NSCs demonstrated an increase in the number of cells over the measured time course across all conditions (Figure 5B). Calculations of F-actin produced per cell showed a relatively consistent trend over the time course (Figure 5C). While NSCs exhibited 60–70% positivity for the proliferation marker Ki67 across all culture conditions early on (Figure 5D), a universal reduction in Ki67^+^ cells was subsequently observed across all conditions, suggesting that terminal differentiation had likely begun (Figure 5D). These results are consistent with previous *in vitro* 3D culture models demonstrating a reduction in Ki67 expression in the early stages of differentiation.^39^ Our data demonstrate that cells cultured on uncoated or PLO+laminin-coated microsphere scaffolds display characteristics comparable to those of cells exposed to the poorly defined supportive effects of Matrigel.

### A Scaffold-Based Model Supports Neural Differentiation of Both Control and Patient-Derived iPSC Models

**Figure 6:**
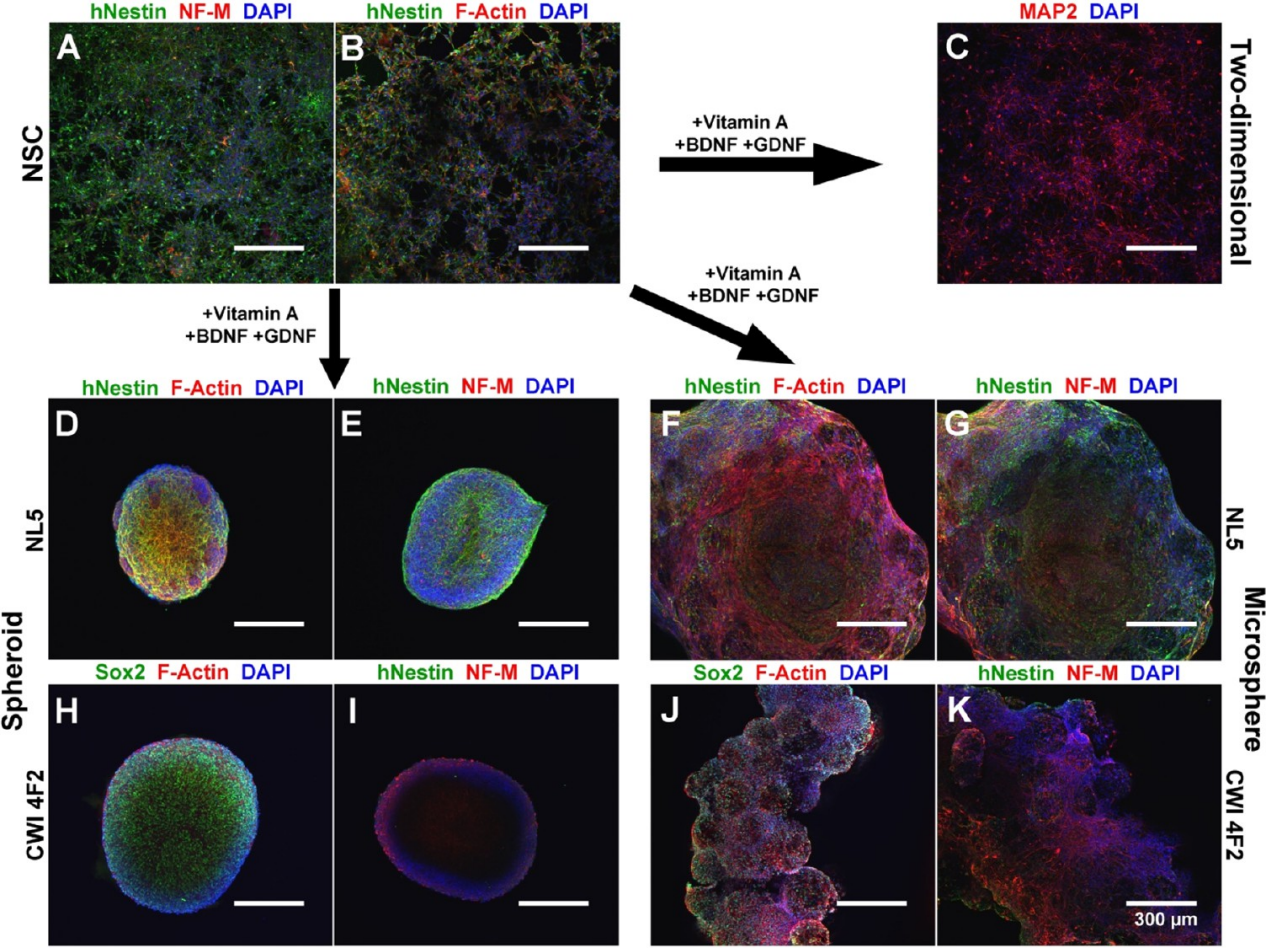
Microsphere-cultured control and patient-derived iPSC derivatives exhibit early neuronal lineage commitment. Comparisons of control (NL5) and patient (CWI 4F2) models in 2D and 3D neurospheres and 3D microspheres after 7 days differentiation are shown. (A, B) NSCs exhibit low amounts of NF-M and F-actin but abundant hNestin expression. (C) 2D differentiation produces extensive MAP2 expression. (D, E) Control NSCs cultured as scaffold-free neurospheres labeled by ICC for (D) hNestin, F-actin, and DAPI or (E) hNestin, NF-M, and DAPI. (F, G) Control NSCs cultured as cellular scaffolds labeled by ICC for (F) hNestin, F-actin, and DAPI or (G) hNestin, NF-M, and DAPI. (H, I) CWI 4F2 NSCs cultured as a scaffold-free neurosphere labeled by ICC for (H) Sox2, F-actin, and DAPI or (I) hNestin, NF-M, and DAPI. (J, K) CWI 4F2 NSC scaffolds labeled by ICC for (J) Sox2, F-Actin, and DAPI or (K) hNestin, NF-M, and DAPI. Scale bars = 300 μm.

Recent studies have questioned the quality of 2D monolayer neural culture because of the inability of cells to become polarized on rigid, flat surfaces.^13^ To evaluate the differentiation of iPSC-derived NSCs cultured on a microsphere scaffold relative to traditional differentiation models, we compared the differentiation of control and patient-derived iPSCs within a two-dimensional system, as self-aggregating neurospheres, and cultured on microsphere scaffolds. The CWI 4F2 patient iPSC line is a model for the cholesterol synthesis disorder Smith–Lemli–Opitz syndrome, a rare disease where subjects exhibit significant neurological malformations.^29,40^ We previously demonstrated that this cell line exhibits stem cell defects and accelerated neuronal differentiation.^29^ After 7 days of differentiation, we verified the multilineage differentiation of both control and patient-derived NSCs using ICC. Cultured cell lines exhibited extensive expression of the human neural progenitor marker hNestin, the pan-neuronal marker βIII-tubulin, the neuronal dendritic marker microtubule-associated protein-2 (MAP2), and the astrocyte marker glial fibrillary acidic protein (GFAP) (Figure 6A–C). Compared with traditional 2D culture, spheroid culture allowed for abundant hNestin+ neural progenitors but very little NF-M expression (Figure 6D,E). In scaffold-based culture, control (NL5) NSCs showed abundant hNestin expression, F-actin, and high expression of NF-M (Figure 6F,G). CWI 4F2 patient neurospheres exhibited both high levels of Sox2 and NF-M compared with NL5, in agreement with the previously published accelerated neuronal differentiation phenotype in this model (Figure 6H,I).^29^ In comparison, CWI 4F2-cultured scaffolds demonstrated a mixed neural lineage, including Sox2^+^ and hNestin^+^ NSCs as well as extensive NF-M expression (Figure 6J,K). Analysis of F-actin also demonstrated increased cytoskeleton formation within both control and patient-derived cells on scaffold versus neurospheres (Figure 6D,F,H,J).

**Figure 7:**
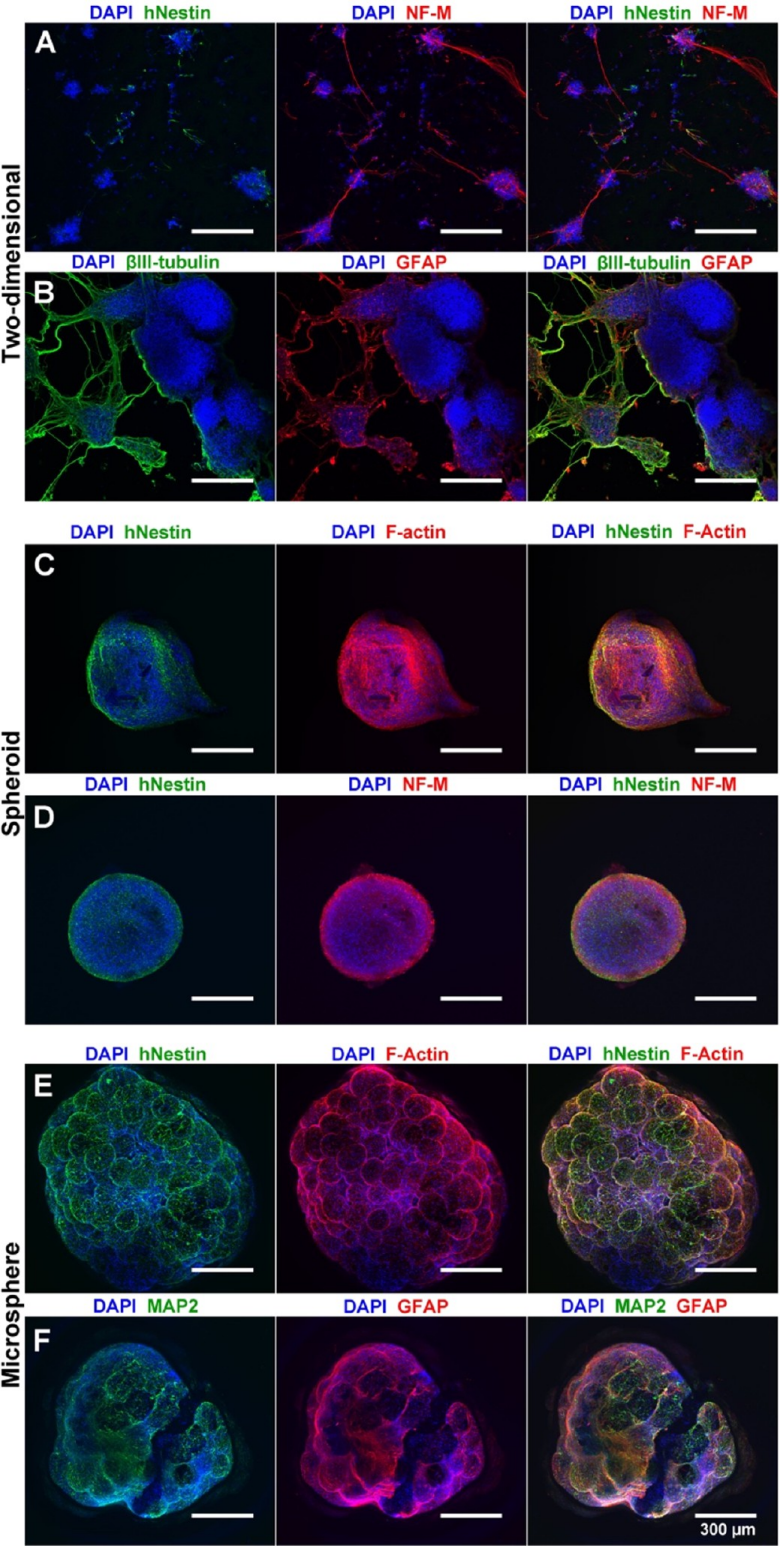
Microsphere culture allows NSC differentiation to neuronal and glial lineages. (A, B) 28 day differentiation under 2D conditions generates extensive neuronal (NF-M, βIII-tubulin) and astrocyte (GFAP) formation with loss of NSCs (hNestin) (DAPI nuclear counterstain). (C, D) Spheroid culture maintains NSCs (hNestin) over 28 days with neuronal (NF-M) and cytoskeletal (F-actin) formation (DAPI nuclear counterstain). (E, F) Microsphere culture allows for expansion and cytoskeletal production of NSCs (hNestin, F-actin) as well as robust differentiation to neuronal (MAP2) and astrocytic (GFAP) lineages. Scale bars = 300 μm.

After 28 days of differentiation, NSCs cultured on 2D PLO+laminin-coated coverslips underwent considerable morphological change. While extensive GFAP^+^ astrocytes were observed by day 28, differentiated neurons formed cell clumps and demonstrated loss of cell adhesion associated with diminished cell health (Figure 7A,B). While spheroid cultures maintained a uniform cell distribution and overall structure, the spheroid size was unchanged compared to day 7. Furthermore, spheroids exhibited increased NF-M^+^ neurons compared with day 7 while maintaining high F-actin and hNestin levels (Figure 7C,D). However, GFAP expression was not observed in spheroids. In comparison to spheroid cultures, the diameter of the microsphere scaffold cultures was significantly increased on day 28 of differentiation (Figure 7E,F). Scaffold-based cultures also demonstrated extensive glial differentiation, as exhibited by GFAP^+^ cell types. The expansive glial differentiation within scaffold-based cultures did not occur at the expense of neuronal differentiation, as evidenced by extensive MAP2 expression. Using immunohistochemistry, we further determined that scaffold-based cultures exhibited integration of NSC-derived cells throughout the microsphere at day 56 (Figure S2). H&E staining demonstrated broad distribution of cells throughout the scaffold, validating the ability of cells to migrate from the scaffold’s exterior surface. Overall, these assays demonstrate that our microsphere matrix provides a chemically defined, neural-supportive microenvironment that allows expansion, migration, and multilineage differentiation of both control and patient-derived NSCs.

**Figure 8:**
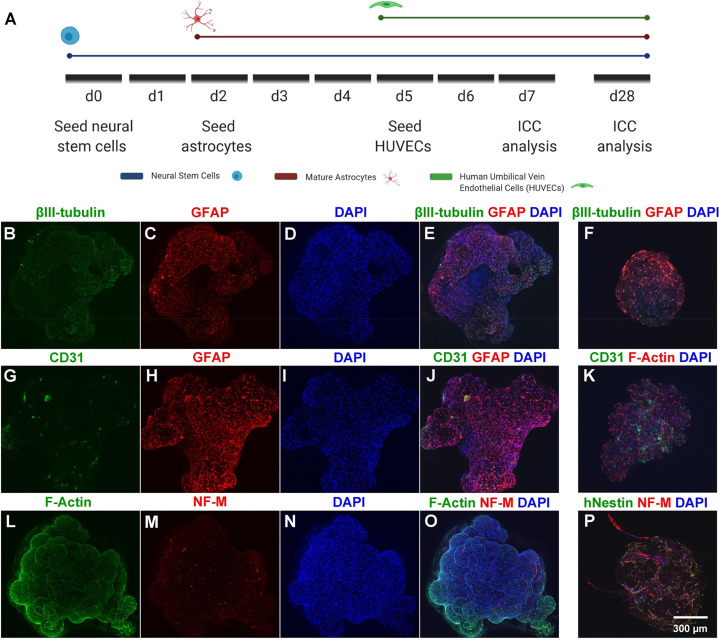
Microsphere-based scaffolds support the coculture of NSCs, astrocytes, and HUVECs. (A) Cell seeding and ICC analysis timeline. Confocal images are displayed as maximum projections of iPSC-derived NSCs, astrocytes, and HUVECs within the scaffold. (B–E) Day 7 ICC for neurons (βIII-tubulin) and astrocytes (GFAP). (F) Day 28 ICC for neurons (βIII-tubulin) and astrocytes (GFAP). (G–J) Day 7 ICC for HUVECs (CD31) and astrocytes (GFAP). (K) Day 28 ICC for HUVECs (CD31) and cytoskeletal formation (F-actin). (L–O) Day 7 ICC for cytoskeletal formation (F-actin) and mature neurons (NF-M). (P) Day 28 ICC for neural progenitors (hNestin) and mature neurons (NF-M). DAPI nuclear counterstain is also shown. Scale bars = 300 μm.

Recent work has demonstrated that coculture of endothelial cells with iPSC-derived models supports neural health and maturation.^41,42^ To demonstrate the capacity of our scaffold-based system for multilineage coculture, NSCs, astrocytes, and endothelial cells were sequentially seeded onto a PLO+laminin-coated microsphere scaffold. NSCs were first seeded onto microspheres in ultralow-attachment 96-well plates, followed by astrocytes and finally HUVECs (Figure 8A). As demonstrated by expression of βIII-tubulin, GFAP, and CD31 on day 7 of coculture, the scaffold allows for attachment, survival, and integration of each cell type (Figure 8). F-actin expression (as identified by phalloidin–Texas Red) and nuclear counterstaining demonstrated broad cell distribution and cytoskeletal formation throughout the microsphere-based scaffold (Figure 8L,O). ICC demonstrated that astrocytes, neurons, and HUVECs were still identifiable within the cellular scaffold on day 28 of coculture (Figure 8F,K,P). Increased expression of NF-M, GFAP, and CD31 on day 28 suggests increased neuronal maturation and proliferation of astrocytes and HUVECs (Figure 8F,K,P). Further, the maintenance of hNestin+ cells at day 28 suggests continued NSC maintenance within this coculture scaffold. These data further demonstrate the ability of the microsphere scaffold for robust coculture of neural, glial, and endothelial cells, representing a critical initial step toward the formation of mature, nutrient-rich, and vascularized 3D structures using this material.

### Microspheres Can Function as a Platform for Sustained Growth Factor Release

**Figure 9:**
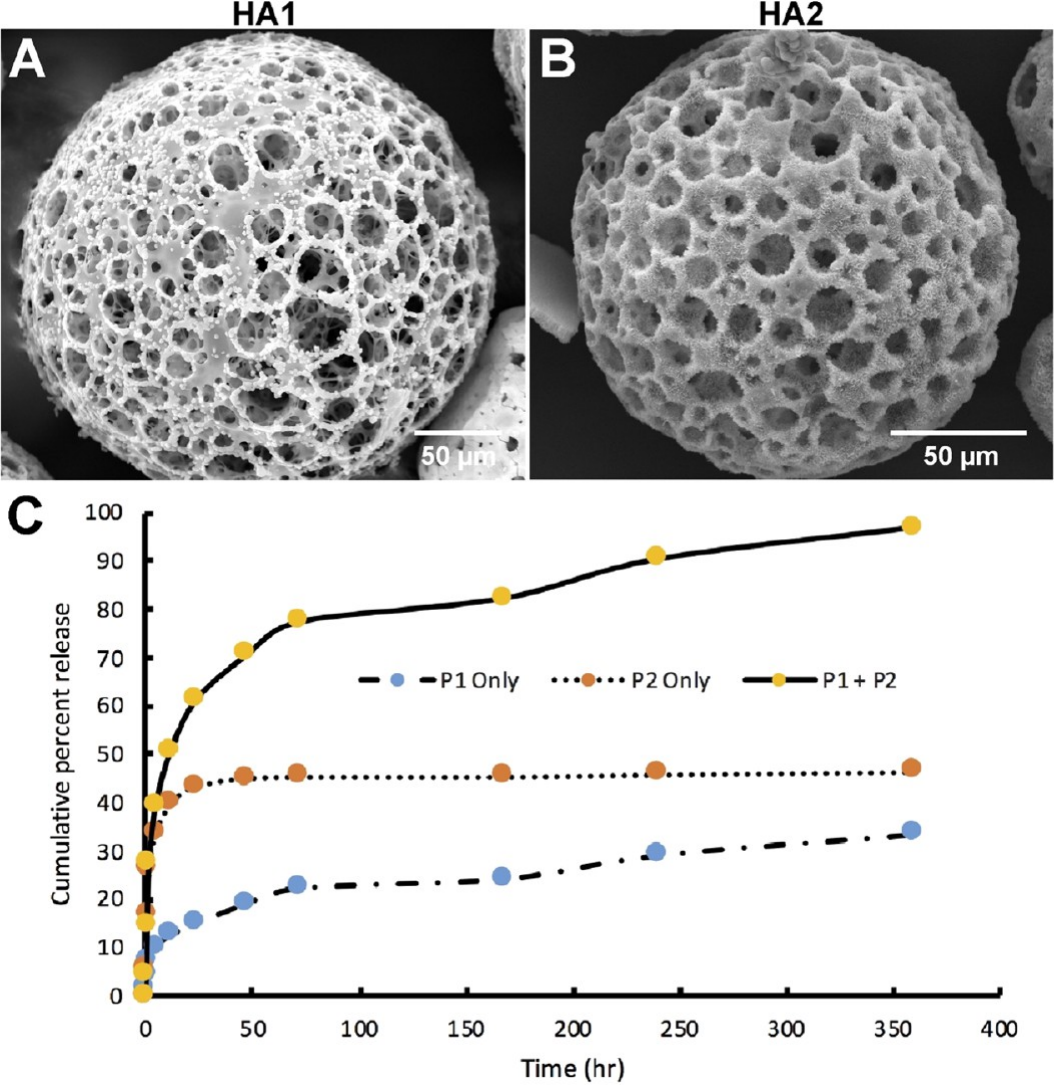
Hydroxyapatite-coated microspheres allow for protein loading and release. (A) SEM image of a PLGA microsphere covered in hydroxyapatite nucleation crystals after immersion in SBF phase I (P1) solution. Scale bar = 50 μm. (B) SEM image of a PLGA microsphere covered in mature hydroxyapatite crystals after immersion in SBF phase 2 (P2) solution. Scale bar = 50 μm. (C) Greater amounts of BSA were released from P1+P2 compared to P1 only or P2 only after 360 h in solution (*n* = 4).

Neural differentiation of iPSCs requires frequent exogenous supplementation of defined cocktails of growth factors and cytokines to promote cell proliferation, differentiation, and tissue organization. To determine whether microspheres could function in both cellular support and growth factor release, microspheres were layered with hydroxyapatite crystals *via* SBF. While hydroxyapatite has traditionally been utilized for osteogenic differentiations,^43,44^ recent work has demonstrated that hydroxyapatite also promotes neural differentiation and functional neuronal development through enhanced Ca^2+^ signaling.^45^ HA was deposited onto the entire exposed exterior and interior surfaces of the microsphere, allowing crystal deposition without pore occlusion (Figure 9A,B). The first SBF phase deposited on the microsphere surface (HA1) acts as a nucleation site, while deposition of the second phase creates an additional layer (HA2) (Figure 9A,B). To model the capacity of the two HA layers to entrap and release proteins, BSA was added to SBF phase 1 and phase 2 solutions. BSA entrapment was evaluated in three different combinations: BSA added to SBF phase I only (P1 only), BSA added to SBF phase 2 (P2 only), or BSA added to both SBF phases (P1+P2). While for P1 only the incorporation of BSA was relatively inefficient (7.2%), P2 only (34.5%) and P1+P2 (56.3%) demonstrated robust protein incorporation into HA layers. BSA release following P1+P2 entrapment was also highly efficient (96.9 ± 3.56%) (Figure 9C). The release rates among the three groups varied relative to the observed incorporation efficiency. The P1 only group (7.2% incorporation efficiency) had an overall release rate of 0.008 μg/min during the 360 h release time frame. In comparison, the P2 only group (34.5% incorporation efficiency) and the P1+P2 group (56.3% incorporation efficiency) had overall release rates of 0.04 and 0.14 μg/min, respectively, over the 360 h time frame.

**Figure 10:**
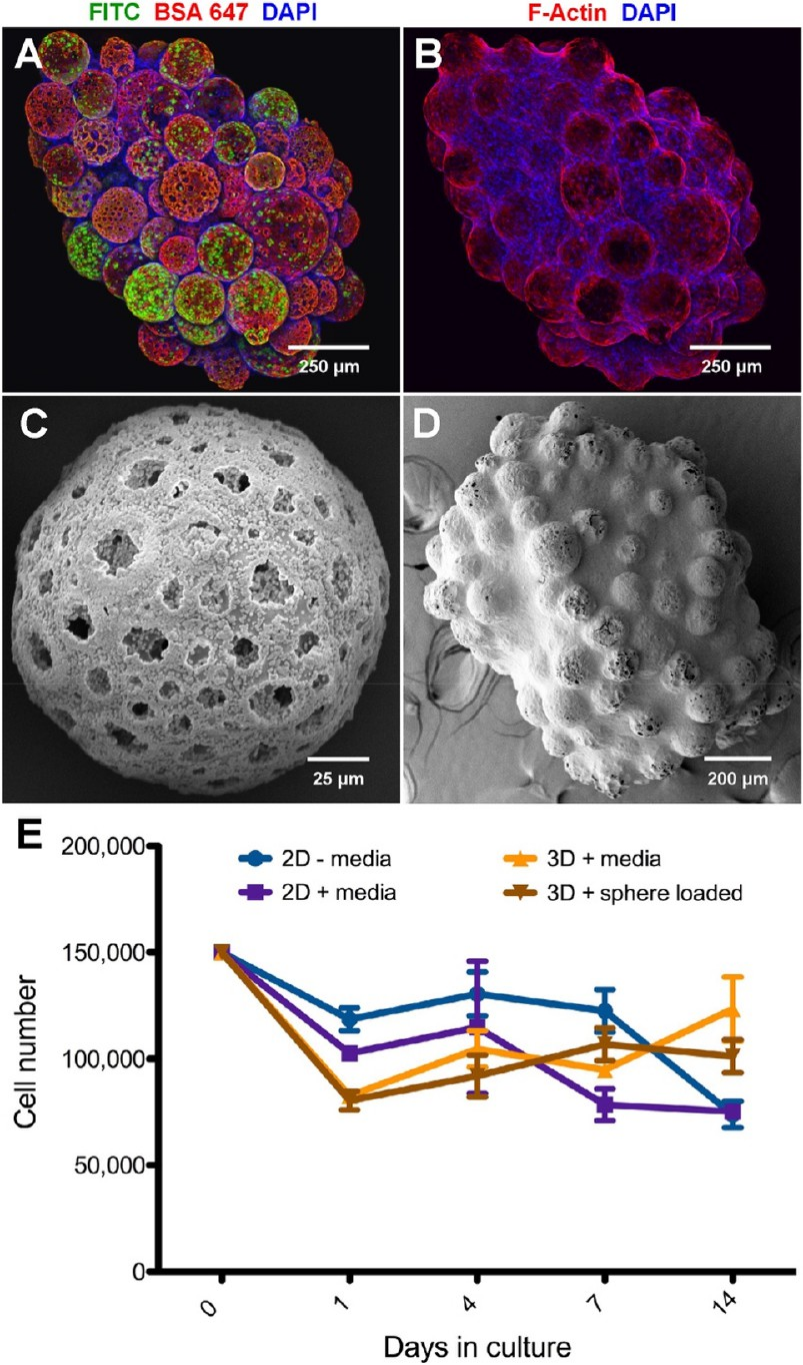
Protein-loaded and hydroxyapatite-coated microspheres supply growth factors directly to scaffold-cultured NSCs. (A) Confocal images of NSCs on an HA-microsphere-based scaffold with merged ICC channels showing FITC–peptide in phase I HA, BSA–Alexa Fluor 647 in phase II HA, and cell nuclei counterstained with DAPI. Scale bar = 250 μm. (B) Confocal images of NSCs on an HA-microsphere-based scaffold with merged ICC channels showing F-actin filaments identified with phalloidin–Texas Red and nuclei counterstained with DAPI. Scale bar = 250 μm. (C) SEM image of a microsphere with bFGF incorporated into the HA matrix (P1+P2). Scale bar = 25 μm. (D) SEM image of an NSC-cultured microsphere scaffold after 5 days. The scaffold contains bFGF incorporated into HA. Scale bar = 200 μm. (E) bFGF released directly from HA promoted NSC proliferation over 14 days comparably to bFGF-supplemented medium. Error bars represent ±1 standard deviation.

After verifying that an entrapped protein could be loaded and released in a controlled and sustained manner, we sought to determine whether the scaffold could support loading and release of multiple molecules. Two biomolecules were loaded into hydroxyapatite-coated microspheres: a FITC-conjugated peptide was loaded into phase 1 HA, and Alexa Fluor 647-conjugated BSA was incorporated into phase 2 HA. NSCs were seeded onto the scaffold following protein entrapment, followed by imaging for FITC, Alexa Fluor 647, and DAPI-counterstained NSC nuclei (Figure 10A). NSCs attached onto the surfaces of all HA-coated microspheres and formed robust cytoskeletal projections across the scaffold (Figure 10B). To determine the bioactivity of entrapped biomolecules, bFGF was entrapped in both phases of the HA crystal matrix (P1+P2). The loading of bFGF into both HA layers did not interfere with the porous structure of the microsphere, as the microsphere matrix was covered in HA crystals (Figure 10C). While bFGF-loaded crystals appeared somewhat flattened compared to HA crystals without loading (Figure 10D), the bFGF-entrapped scaffold demonstrated increased NSC proliferation in scaffold cultures compared with standard 2D culture (Figure 10E). These data demonstrate that the microsphere scaffold can be utilized for entrapment and release of proteins of interest in a sustained manner, providing direct trophic factor support to seeded cells.

## Discussion

PLGA has been widely used as a biomaterial to support and direct cell fate through various 3D tissue engineering scaffold fabrication techniques such as electrospinning, soft lithography, gas foaming, particle leaching, supercritical CO_2_, phase separation, 3D printing, and freeze-drying.^46−50^ Polymeric and composite materials utilizing PLGA have been used to align tenocytes to support tendon repair, induce chondrogenesis of rabbit mesenchymal stem cells, and promote hepatogenesis of human adipogenic stem cells and differentiation of canine smooth muscle cells.^47,51^ PLGA scaffolds were used with and without the addition of transforming growth factor-β3 to support the delivery and differentiation of mesenchymal stem cells toward articular cartilage *in vivo*.^52^ Our work has demonstrated that PLGA microspheres provide a multifunctional 3D cell culture platform that is also capable of loading and releasing proteins, peptides, and other growth factors. By incorporating biocompatible materials, using defined starting numbers of stem cells, and providing a chemically defined environment, our scaffold platform addresses some of the current challenges limiting the utility of 3D cell culture.^10^ The microsphere scaffold developed here can be readily produced in high numbers, the product is shelf-stable for future use, and the final microsphere diameter is tunable during preparation. We have further demonstrated that this system can be used to allow effective neural differentiation in three dimensions. Though the Young’s modulus of PLGA is higher than that of the presumptive ECM of the brain, substrate stiffness differs between areas of the brain and within glial subtypes. Studies have reported a stiffness range from 0.1 to 16 kPa across brain regions.^1,7,53^ Substrate stiffness also influences neural subtype differentiation. Neuronal differentiation favors softer substrates (100–500 Pa), while stiffer substrates (1–10 kPa) favor glial differentiation.^1,53^ Rat NPCs cultured on surfaces with stiffnesses of up to 35 kPa were not affected by the discrepancy with native tissue stiffness.^1^ Despite having a higher elastic modulus in its dry state, PLGA undergoes bulk degradation through hydrolytic cleavage of ester bonds along the polymer backbone as water penetrates the matrix.^54,55^ As our work confirms, PLGA was previously shown to soften over the first 48 h as the result of a 221–350% increase in water content.^55,56^ Previous work with PLGA has demonstrated a significant reduction of the elastic modulus due to matrix swelling and rapid loss of molecular weight through the bulk degradation process.^54,56^ In our study, hydration of PLGA microspheres reduced the elastic modulus by approximately 4-fold. The microspheres used here were designed to be a malleable substrate that softens and degrades, allowing for cell remodeling and migration.^12^

The undefined ECM and growth factor milieu of naturally derived hydrogels exposes self-aggregating and self-organizing cells to a poorly controlled mix of excitatory, proliferative, instructive, mechanotransducive, and inhibitory signals.^12,38^ Matrigel-based methods can result in low reproducibility and poor control of differentiation due to the inherent variability within Matrigel.^4,57^ The use of a chemically undefined environments may also obscure or limit the utility of observations.^4,12,53,58,59^ The use of serum-free formulations has created more defined and consistent neural differentiation methods.^6,60^ Therefore, a more defined 3D structure that incorporates neural ECM components would be a beneficial differentiation platform. Through incorporation of substrate-specific matrixes such as PLO+laminin, this study offers improved control over the *in vitro* microenvironment by providing physiologically relevant cues found in the brain.^1,6,47,61,62^ We have demonstrated that the microspheres promote iPSC-derived NSC growth and differentiation. Compared with cell-only 3D neurospheres, which rely on cell aggregation, cell-secreted ECM proteins, and self-organization to generate the 3D structure, the microspheres can be coated with ECM proteins and ligands to mechanically and chemically direct stem cell differentiation. Scaffolds with high porosity and nearly 100% interconnected pore structure, such as the microsphere platform presented here, allow nutrients, oxygen, and waste products to be transported throughout the biomaterial-based organoid structure.^1,63^ We have modeled the flow of solution through the microsphere by the deposition of HA crystals throughout the internal architecture of the microsphere. The larger surface area, porosity, and biocompatibility of PLGA microspheres support cell attachment, growth and differentiation.^4,20,64^ The acidic byproducts that form upon matrix degradation can lower the pH and lead to inflammation within PLGA-based scaffolds.^55,65^ However, less than 12% of our microsphere volume is composed of PLGA. Furthermore, the interconnected pore structure allows lactic acid and glycolic acid monomers to be diluted within the surrounding medium, limiting toxicity toward scaffold-based cells.^47,51,66^ Finally, the porous matrix and the high surface area of the scaffolds create a supportive environment that promote cellular health and complexity compared with cell-based neurospheres.

Because of the frequent inability of animal models to recapitulate disease manifestation,^1,11^ the ability to model human disease using iPSCs in a 3D environment is critical for both basic and translational research. The ability to model human disease *in vitro* with iPSCs allows access to both unaffected and disease-impacted cell types of interest, providing opportunities for analysis of disease pathogenesis or drug discovery studies.^2^ However, the cellular complexity of iPSC-based neurological models has been limited by the stochastic nature of the differentiation process. We have demonstrated that our 3D-microsphere-based scaffold system can function as an *in vitro* neurodevelopment platform using iPSC-derived cells. Our system can support both unaffected and disease-affected iPSC models as well as combinatorial culture of progenitors, differentiated neuronal and glial cell types, and endothelium.^1,37,67,68^^29^

While we have demonstrated that our microsphere platform can successfully host cell types of interest, future studies utilizing this platform will determine the functional activity of cultured cells, the impact of cell-to-cell interactions, the optimization of cell populations, and the utilization of ECM coatings favorable to specific cell types. Such studies will involve prolonged (multimonth) culture to allow maturation and functional development of cellular networks, as has previously been performed in self-organizing cerebral organoid models.^69^ Through directed differentiation toward specific cell types of interest in separate scaffolds, the microspheres could be combined, similar to assembloids, to create composite scaffolds with greater heterogeneity and functionality. The microsphere-based scaffold architecture offers a unique platform to assemble distinct clusters of differentiating cells to maximize recapitulation of central nervous system regions of interest. Future studies will therefore be needed to determine the precise impact of our microsphere scaffold on the formation and function of defined neuronal and glial populations.

Our data demonstrate that the microsphere platform described here can function as both a cellular scaffold and a growth factor elution system consisting of biocompatible materials. This work provides important proof-of-concept data regarding the multifunctionality of this system. The HA-coated microspheres described here can be loaded with multiple growth factors, as demonstrated by incorporation of two fluorescently bound molecules. Future work will evaluate other bioactive molecules, such as silk nanofibers, which limit substrate stiffness compared to HA for the incorporation and release of soluble factors.^70^ The incorporation of physiologically critical growth factors, such as bFGF, into a 3D platform has the capacity to promote progenitor proliferation or drive cellular differentiation without additional environmental manipulation. Proteins, peptides, and other small molecules can thus be released directly to cells to modify a signaling pathway or cellular function without disturbing the growing organoid. The porous structure allows for a much greater loading capacity due to the surface area, as well as rapid clearance of any acidic byproducts that may interfere with the bioactivity of sensitive molecules.^55^ We have demonstrated that bFGF released from the microspheres over 14 days increased proliferation above the level of the 2D monolayer that received bFGF-supplemented medium every other day. In a similar manner to coating microspheres with various proteins to model different ECM substrates, the microspheres can be dual-loaded with factors to influence attached cells. For example, the addition of a bioceramic component to PLGA microspheres is applicable for use in other, non-neural tissue engineering models.

## Conclusion

We have developed a chemically defined, microsphere-based cell culture platform to model neurodevelopment and disease pathogenesis using iPSC derivatives. The microspheres developed in this study represent a biodegradable, highly porous, customizable substrate capable of hosting NSCs and differentiated cell types for weeks *in vitro*. We have shown that the platform can be customized with various extracellular matrixes such as PLO and laminin to support proliferation or directed differentiation, as desired. We have further demonstrated that these microspheres can support multiple neural and non-neural cell types simultaneously through coculture of NSCs, NSC-differentiated neurons, mature astrocytes, and HUVECs. Finally, the modified microspheres can simultaneously function as both a cellular scaffold and a small-molecule delivery platform. Future work will use the biophysical and nanoarchitectonic cues utilized here to generate complex culture systems for the study of development, disease pathogenesis, or 3D-based drug discovery assays.

## Supporting Information Available

Additional data and figures describing FTIR analyses of nanoarchitectonic-derived microsphere components, generated microspheres in the presence or absence of HA coating, and H&E imaging of cellular distribution within the microsphere following long-term culture (PDF)

## Notes

Datasets generated in this study are available from the corresponding author upon request.
