# Supplementary material for: Nanoarchitectonics of a Microsphere-Based Scaffold for Modeling Neurodevelopment and Neurological Disease
Source: ACS Appl Bio Mater. 2022 Jan 19;5(2):528–44. doi: 10.1021/acsabm.1c01012 (PMC8865216; doi:10.1021/acsabm.1c01012)
Supplement: Supplementary file 1 — mt1c01012_si_001.pdf [file mt1c01012_si_001.pdf]

Supporting Information

Nanoarchitectonics of a Microsphere-Based Scaffold for Modeling Neurodevelopment and Neurological Disease

Eric S. Sandhurst[a,b], Sharad V. Jaswandkar[c], Krishna Kundu[c], Dinesh R. Katti[c], Kalpana S. Katti[c], Hongli Sun[a,b#], Daniel Engebretson[a], Kevin R. Francis[a,b,d,e*]

[a] Department of Biomedical Engineering, University of South Dakota, Sioux Falls, SD, 57107, USA.

[b] BioSystems Networks and Translational Research Center, Brookings, SD, 57006, USA.

[c] Civil, Construction and Environmental Engineering Department, North Dakota State University, Fargo, ND, 58108, USA.

[d] Cellular Therapies and Stem Cell Biology Group, Sanford Research, Sioux Falls, SD, 57104, USA.

[e] Department of Pediatrics, University of South Dakota Sanford School of Medicine, Sioux Falls, SD, 57105, USA.

[*] Corresponding author: K. R. F. (kevin.francis@sanfordhealth.org)

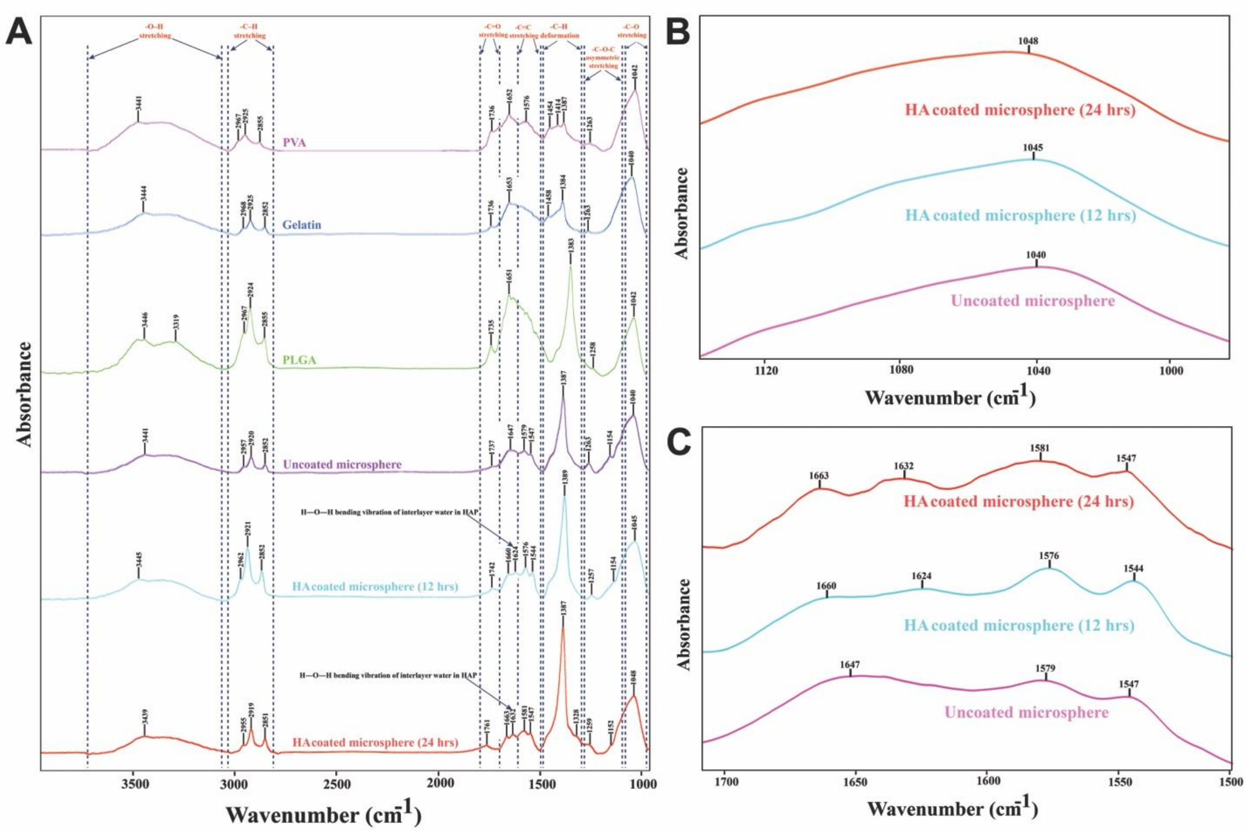

Figure S1. FTIR analysis of the microsphere chemical structure. (A) FTIR spectra of PVA, gelatin, PLGA, microspheres, microspheres coated with HA for 12 h, and microspheres coated with HA for 24 h. The spectra for microspheres coated with HA for 12 h exhibit -OH stretching vibrations (3000 – 3650 cm^{-1}), -CH, -CH2, -CH3 stretching vibrations (2850 – 3000 cm^{-1}), carbonyl C=O stretching vibrations (1700 – 1750 cm^{-1}), a O-hydroxyl band due to intramolecular hydrogen bonding (1663 cm^{-1}), -CH deformation vibrations (1300 – 1500 cm^{-1}), C–O-C stretching vibrations (1300 – 1200 cm^{-1}) and C–O stretching vibrations (1045 cm^{-1}). A band shift from 1040 cm^{-1} to 1045 cm^{-1} occurred due to interaction of HA with C-O. A band at 1624 cm^{-1} due to the H—O—H bending vibration of interlayer water in HA was also observed. Microspheres coated with HA for 24 h exhibit -OH stretching vibrations (3000 – 3650 cm^{-1}), -CH, -CH2, -CH3 stretching vibrations (2850 – 3000 cm^{-1}), carbonyl C=O stretching vibrations (1700 – 1780 cm^{-1}), intramolecular hydrogen bonding of O-hydroxyl band (1660 cm^{-1}), -CH deformation vibrations (1300 – 1500 cm^{-1}), C–O-C stretching vibrations (1300 – 1200 cm^{-1}) and C–O stretching vibrations (1048 cm^{-1})[93]. (B, C) Comparison of spectra between uncoated, 12 h HA coated, and 24 h HA coated microspheres. A band shift from 1040 cm^{-1} to 1048 cm^{-1} due to interaction of HA with C-O was identified. We also observed bands at 1624 cm^{-1} in 12 h coated microspheres and 1632 cm^{-1} in 24 h coated microspheres resulting from H—O—H bending vibration of interlayer water in HA.

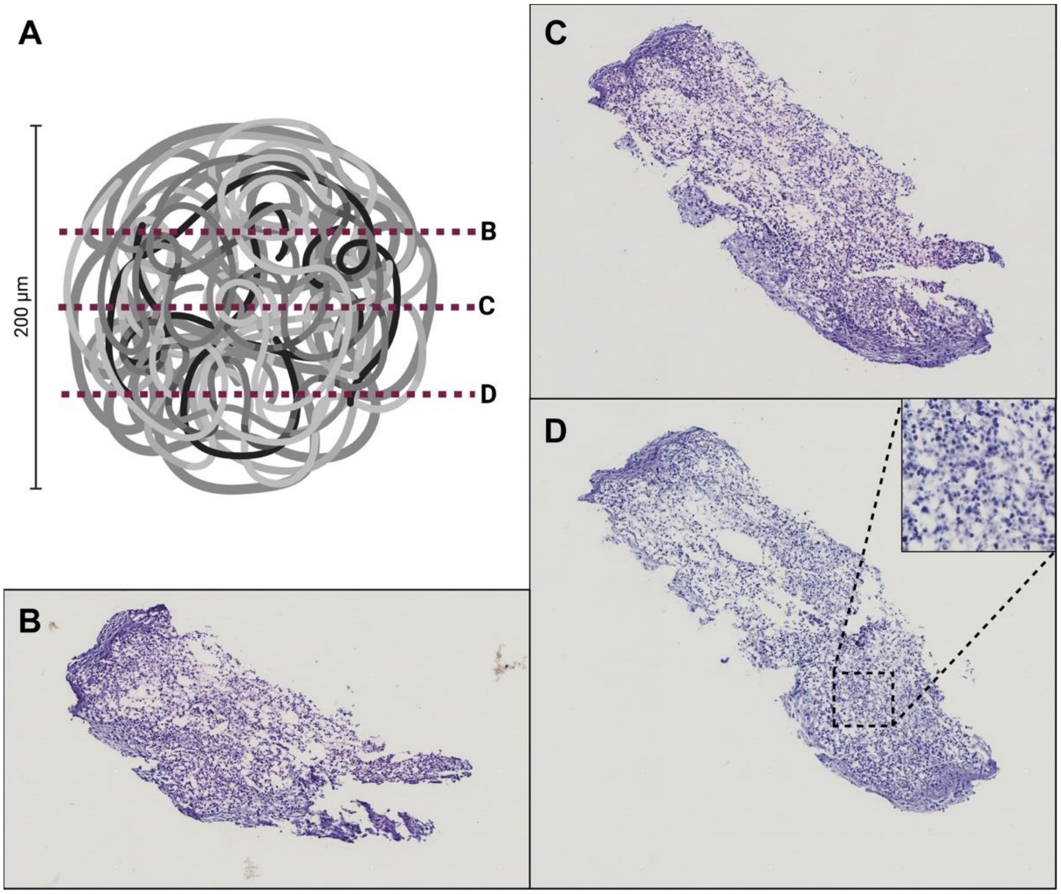

Figure S2. Neural stem cell derivatives exhibit broad distribution within the entirety of the three-dimensional microsphere scaffold. (A) d56 microsphere cultures (~200 μm in diameter) were paraffin embedded, sectioned, and analyzed by H&E immunohistochemical stain for nuclear identification. (B-D) Representative H&E staining of nuclei at multiple cross-section depths within microsphere cultures. Nuclei were easily identified in cross-sections taken approximately 60 μm (B), 110 μm (C), and 165 μm (D) from the surface of the scaffold. Inset in (D) demonstrates high cellularity within the internal microsphere structure.
